# The Potential of the Cyclotide Scaffold for Drug Development

**DOI:** 10.3390/biomedicines7020031

**Published:** 2019-04-19

**Authors:** Julio A. Camarero, Maria Jose Campbell

**Affiliations:** 1 Department of Pharmacology and Pharmaceutical Sciences, University of Southern California, CA 9033, USA; mariajos@usc.edu; 2 Norris Comprehensive Cancer Center, University of Southern California, Los Angeles, Los Angeles, CA 9033, USA

**Keywords:** cyclotides, CCK, cystine-knot, drug design, backbone cyclized polypeptides, protein-protein interactions, cyclic peptides

## Abstract

Cyclotides are a novel class of micro-proteins (≈30–40 residues long) with a unique topology containing a head-to-tail cyclized backbone structure further stabilized by three disulfide bonds that form a cystine knot. This unique molecular framework makes them exceptionally stable to physical, chemical, and biological degradation compared to linear peptides of similar size. The cyclotides are also highly tolerant to sequence variability, aside from the conserved residues forming the cystine knot, and are orally bioavailable and able to cross cellular membranes to modulate intracellular protein–protein interactions (PPIs), both in vitro and in vivo. These unique properties make them ideal scaffolds for many biotechnological applications, including drug discovery. This review provides an overview of the properties of cyclotides and their potential for the development of novel peptide-based therapeutics. The selective disruption of PPIs still remains a very challenging task, as the interacting surfaces are relatively large and flat. The use of the cell-permeable highly constrained polypeptide molecular frameworks, such as the cyclotide scaffold, has shown great promise, as it provides unique pharmacological properties. The use of molecular techniques, such as epitope grafting, and molecular evolution have shown to be highly effective for the selection of bioactive cyclotides. However, despite successes in employing cyclotides to target PPIs, some of the challenges to move them into the clinic still remain.

## 1. Introduction

The selective disruption of pharmacologically relevant protein–protein interactions (PPIs) still remains a very challenging task [1,2,3]. This is mostly due to the relatively large and relatively flat nature of the binding surfaces involved in most PPIs. The most challenging of the molecular targets are in fact those involving intracellular PPIs, which, in addition, require the therapeutic agent to be able to cross the cell membrane in an efficient manner [4,5]. 

In general, we can consider two major structural types of therapeutic agents, small molecules and protein-based compounds, with the later also known as biologicals. Small molecules, as their name indicates, are small in molecular size (≤100 atoms) and in general show good pharmacological properties, such as stability and cell permeability. Their intrinsic small size, however, only provides a modest overall surface area available to interact with the target protein, making the identification of small molecules able to efficiently disrupt PPIs quite challenging [6,7].

On the other hand, the use of polypeptide-based molecules has been able to provide efficient therapeutic tools to modulate PPIs with high specificity and selectivity [8]. The use of therapeutic monoclonal antibodies to target extracellular protein receptors is just one example [9,10]. Antibodies, however, suffer from clear limitations: they are expensive to produce, cannot be delivered orally, show low tissue penetration, and are unable to reach intracellular targets. These issues have led to the exploration of alternative protein scaffolds as a source for novel protein-based therapeutics [11,12,13,14,15,16].

**Figure 1:**
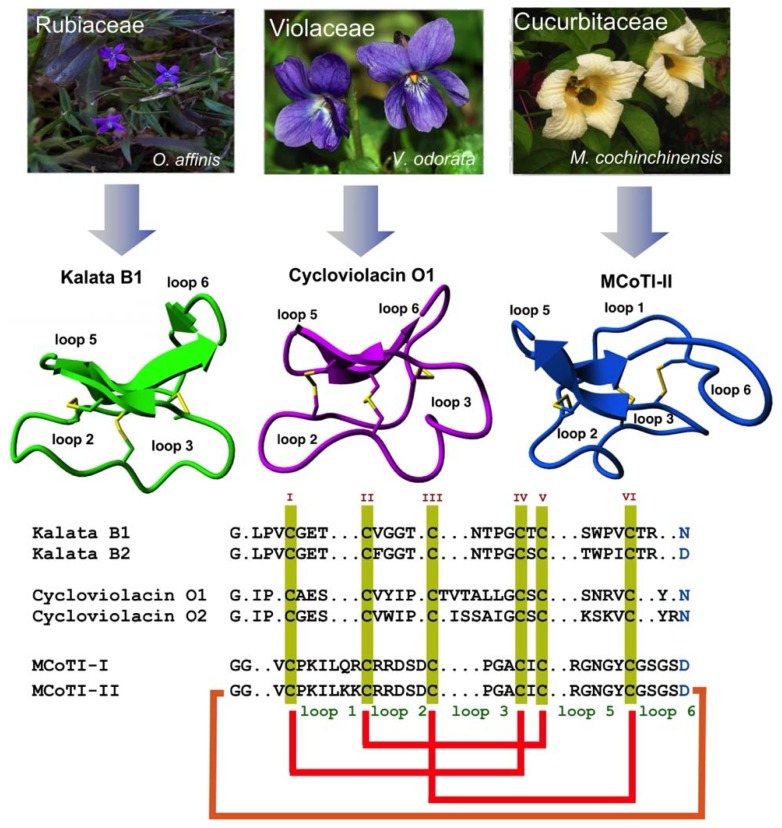
Biological origin, structures, and sequence alignment of different cyclotides belonging to the Möbius (kalata B1, pdb: 1NB1) [24], bracelet (cycloviolacin O1, pdb: 1NBJ) [24], and trypsin inhibitor (MCoTI-II, pdb: 1IB9) [25] subfamilies. These three naturally-occurring cyclotides were isolated from *O. affinis* (*Rubiaceae* family), *Viola odorata* (*Violaceae* family), and *M. cochinchinensis* (*Cucurbitaceae* family). The six Cys residues are labeled with roman numerals, whereas loops connecting the different Cys residues are designated with Arabic numerals. Conserved Cys and Asp/Asn (required for backbone cyclization in nature) residues are marked in yellow and light blue, respectively. Disulfide connectivities and backbone-cyclization are shown in red and orange, respectively. Molecular graphics were created using Yasara (www.yasara.org). Figure adapted from references [17,23].

Special attention has been recently given to the use of highly constrained polypeptides for the development of novel stable polypeptide-based therapeutics [17,18,19]. Cyclotides are a fascinating emerging family of large plant-derived backbone-cyclized polypeptides (≈30–40 amino acids long) containing a 3 disulfide-stabilized core characterized by an unusual knotted structure (Figure 1) [20]. This unusual topology confers the cyclotide scaffold with unique characteristics that make them ideal for drug development (see recent reviews [21,22,23]). 

Cyclotides are remarkably stable to thermal and chemical denaturation and biological degradation by proteolytic enzymes [26]. They can be easily accessible by chemical synthesis due to their relative small size and can be also recombinantly produced using standard expression vectors in different types of cells (see a recent review on the production of cyclotides [27]). Some cyclotides have been shown to be able to cross the cellular membranes of mammalian cells [28,29] to modulate intracellular PPIs, both in vitro and in vivo [5]. Even more exciting, cyclotides have shown to have biological activity when dosed orally [26,30,31]. 

The naturally-occurring cyclotide kalata B1, which was the first cyclotide to be discovered in plants, was used as an orally effective uterotonic [26] and several other kalata B1-based cyclotides have also been shown to be orally bioavailable [30,31]. These unusual characteristics for a polypeptide-based molecular scaffold make the cyclotide molecular framework an ideal substrate for molecular engineering and evolution strategies for the production of novel peptide-based diagnostic, therapeutic, and research tools. This article is meant to provide a brief overview of their most relevant properties and their potential to be used as a molecular scaffold for the development of peptide-based therapeutic agents.

## 2. Structure

All naturally-occurring cyclotides are backbone-cyclized and contain between 27 to 37 amino acids, of which six are Cys residues. The six Cys residues form three disulfide bonds adopt a cystine-knot topology, with disulfides Cys^I^-Cys^IV^ and Cys^II^-Cys^V^ forming a ladder arrangement and disulfide Cys^III^-Cys^VI^ running through it (Figure 1). This highly interlocked cyclic cystine knot (CCK) motif makes the backbone of cyclotides very rigid and compact [32], which is responsible for their high stability to thermal, chemical, and proteolytical degradation [33,34]. This is highlighted in the case of the first cyclotide to be isolated, kalata B1, which was identified in the late 1960s by Gran when studying an indigenous traditional medical remedy in central Africa that was used to facilitate childbirth in pregnant women [35]. This traditional remedy used a tea obtained from the plant *Oldelandia affinis* from the *Rubiaceae* family [36]. The fact that the cyclotide kalata B1 was able to remain folded and biologically active even after being extracted by boiling water to produce a medicinal tea with uterotonic properties shows the remarkable stability of the cyclotide scaffold.

Cyclotides can be classified into three subfamilies, the Möbius, bracelet, and trypsin inhibitor cyclotide subfamilies [37]. All the subfamilies share the CCK topology, however, the loop composition, size, and sequence can be different among the members of the three subfamilies. Cyclotides from the Möbius sub-family, such as kalata B1, have a *cis*-Pro bond at loop 5 formed by a cis tryptophan–proline bond resulting in an 180° twist of the peptide backbone, while bracelet cyclotides do not have it [24]. 

Bracelet cyclotides are the most abundant in nature, making up ≈66% of the all the sequenced cyclotides known thus far [38]. These cyclotides are more structurally different and slightly larger in size than those from the Möbius subfamily. Bracelet cyclotides, on the other hand, are more difficult to fold in vitro than either Möbius or trypsin inhibitor cyclotides, making them more challenging to produce by using standard peptide synthesis protocols [39]. For that reason, cyclotides from the bracelet subfamily are much less used as molecular scaffolds for the production of cyclotides with novel biological activities. 

The third subfamily of cyclotides, the trypsin inhibitor subfamily, contains only a small number of cyclotides isolated from the seeds of several *Momordica spp* plants (*Cucurbitaceae* family) [40,41,42]. Cyclotides from this subfamily are very potent trypsin inhibitors (*K*_i_ ≈ 20 pM) [43] that do not share significant sequence homology with cyclotides from the other two subfamilies beyond the CCK topology common to all cyclotides (Figure 1). Cyclotides from the trypsin subfamily show high sequence homology with cystine knot squash trypsin inhibitors and sometimes are referred to as cyclic knottins [44].

More recently, a new type of cyclotides with high content in positively-charged Lys residues haven also been isolated from two species of Australasian plants from the *Violaceae* family [45]. Unfortunately, there is not yet any information on their chemical synthesis, making it difficult to evaluate their real potential for being used as molecular frameworks in the design of novel peptide-based therapeutics. 

## 3. Biosynthesis

Naturally-occurring cyclotides are produced by enzymatic processing from ribosomally produced precursor proteins [37]. Many cyclotides have dedicated genes encoding multiple copies of the same cyclotide or mixtures of different cyclotide sequences [46]. Not surprisingly, the first dedicated genes encoding cyclotide precursor proteins were found in the cyclotide producing plant *Oldelandia affinis* (*Rubiaceae* family), which is the source of the cyclotide kalata B1 [47,48]. Similar genes have also been found in other plants from the *Violaceae* and *Rubiaceae* [49], and more recently also in plants from the *Solanaceae*, *Fabaceae*, and *Cucurbitaceae* families [20,41,49,50,51]. Some of these new genes provide novel architectures for the corresponding cyclotide protein precursor, showing the high diversity used by nature to generate cyclotides.

Although the post-translational modifications required for the biosynthesis of cyclotides in nature have not been fully characterized yet [49,52], recent studies have determined that asparaginyl endopeptidase (AEP)-like ligases are involved in the C-terminal cleavage and backbone-cyclization of the linear cyclotide precursor [53,54]. For example, the co-expression of a cyclizing AEP-like ligase with a cyclotide precursor protein in non-cyclotide producing plants significantly improves the cyclization efficiency of the corresponding cyclotide linear precursor [55]. These AEP-like ligases have been shown to be able to backbone-cyclize different linear peptides in vitro, including linear cyclotide precursors and even peptides containing D-amino acids [53,55,56,57,58,59]. 

Despite the increasing understanding of how cyclotides are produced in plants, there is still not too much known about the N-terminal cleavage process and the corresponding associated protease. Complete understanding on how the cyclotide linear precursors are processed and identification of all the players involved should facilitate the engineering of genetically-modified organisms for the inexpensive bioproduction of cyclotides [60,61].

## 4. Chemical Synthesis

**Figure 2:**
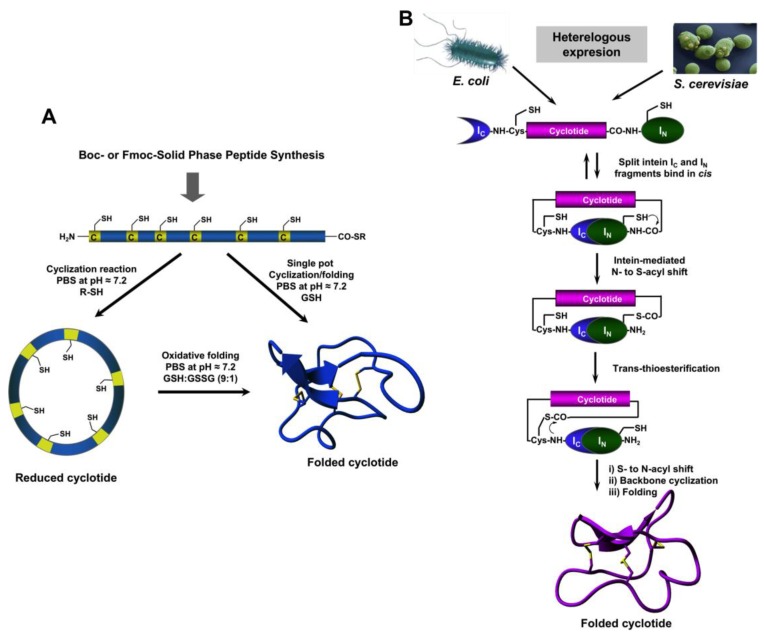
Different available approaches for the production of cyclotides. (**A**) Chemical synthesis of cyclotides by making use of an intramolecular version of native chemical ligation. This approach requires the generation of a linear precursor polypeptide bearing an N-terminal Cys residue and an α-thioester moiety at the C-terminus. The linear precursor can be first cyclized under reductive conditions and then folded using a proper redox buffer, for example using reduced and oxidized glutathione (GSH) [27]. The cyclization and oxidative folding can be also efficiently accomplished in a “single pot” reaction when the cyclization is carried out in the presence of reduced GSH as the thiol cofactor [27]. (**B**) Recombinant expression of cyclotides by making use of the protein trans-splicing (PTS) [73,74,75]. This approach has been employed for the generation of several MCoTI-cyclotides, where the native Cys residue located at the beginning of loop 6 was used to facilitate backbone cyclization. This method can be used to produce bioactive cyclotides in either eukaryotic or prokaryotic expression systems [73,74,75]. Figure adapted from a previous study [23].

The relatively small size of cyclotides makes the synthesis of the corresponding linear precursors by chemical methods possible, using solid-phase peptide synthesis (SPPS) [27]. Backbone cyclization of the linear precursor can be easily accomplished in aqueous buffers at pH ≈7 using an intramolecular version of native chemical ligation (Figure 2A). The required peptide α-thioester can be readily generated using standard solid-phase peptide synthesis methods by either Boc- or Fmoc-based chemistry [27]. The corresponding linear precursor can be cyclized and oxidatively folded sequentially. A very convenient approach to generate chemically-produced cyclotides involves carrying out the cyclization and folding steps in a “single pot” reaction by using glutathione (GSH) as a thiol additive [62]. This approach has successfully been used to chemically generate many native and engineered cyclotides [5,62,63,64], as well as other disulfide-contained backbone-cyclized polypeptides [65,66].

Cyclotide linear precursors can be also chemoenzymatically cyclized using AEP-like ligases [53,54,59,67], which do not require the linear precursor to be natively folded for the cyclization to proceed efficiently [53]. Naturally occurring trypsin inhibitor cyclotides, such as MCoTI-I/II, can also be produced using the serine protease trypsin [68]. This is accomplished by producing a folded linear precursor bearing the P1 and P1 residues at the C- and N-termini, respectively. This approach provides a very efficient route for obtaining cyclotides with trypsin inhibitory properties with yields close to 92% for cyclotide MCoTI-II [68], however the introduction of mutations that affect the binding to the proteolytic enzyme may affect the cyclization yield [27]. Other proteases, such as the transpeptidase like sortase A (SrtA), have been also employed for the backbone cyclization of the corresponding synthetic linear precursor [69]. However, this approach, due to the sequence requirements for SrtA to work properly, leaves an extra heptapeptide motif at the cyclization site, which should be taken into consideration when producing bioactive cyclotides.

## 5. Recombinant Expression

The use of protein splicing units, also called inteins, in either *cis* or *trans* allows the recombinant production of backbone cyclized polypeptides (for more detailed reviews in this topic see [27,70]). Initial attempts for production of cyclotides using heterologous expression systems involved the use of modified inteins for generating α-thioester polypeptides that were then backbone-cyclized using an intramolecular version of native chemical ligation [71,72]. The use of intein-mediated protein *trans*-splicing (PTS) has been shown to be more effective for the production of naturally-occurring and engineered cyclotides in prokaryotic and eukaryotic expression systems (Figure 2B) [73,74,75]. In-cell production of folded cyclotides by PTS can reach intracellular concentrations in the range of 2040– μM. This corresponds to ≈ 10 mg of folded cyclotide per 100 g of wet cells in *Escherichia coli* expression systems producing cyclotide MCoTI-I [75]. These values are quite comparable to those obtained when using the cyclotide-producing plant *O. affinis*, which produces ≈ 15 mg of cyclotide kalata B1 per 100 g of wet weight when grown in vitro [76]. Given the fastest growth rate and the simplicity of working with microorganisms such as *E. coli*, PTS provides a very attractive alternative for a cost-effective route to produce bioactive cyclotides with therapeutic potential.

**Figure 3:**
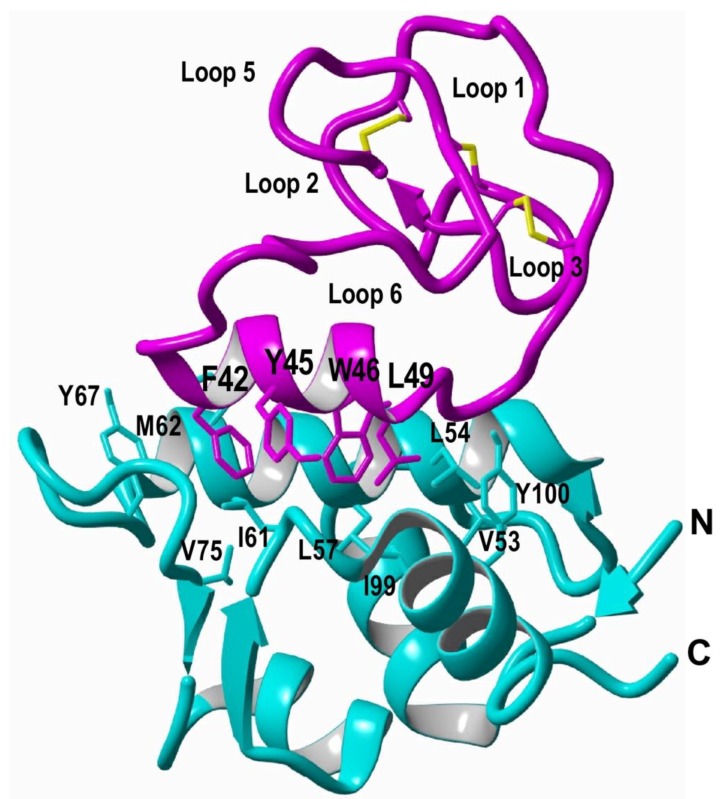
Structure of a MCoTI-based cyclotide designed to antagonize an intracellular PPI [5]. The structure of the engineered cyclotide MCo-PMI (magenta) and its intracellular molecular target, the p53 binding domain of oncogene Hdm2 (blue), were determined in solution by nuclear magnetic resonance (NMR). Cyclotide MCo-PMI binds with low nM affinity to both the p53-binding domains of Hdm2 and HdmX.

In-cell production of cyclotides also opens the exciting possibility for the generation of large genetically-encoded libraries of cyclotides, which can be rapidly screened for the selection of novel sequences able to modulate specific molecular targets [74]. In addition, having easy access to cyclotides using standard heterologous expression systems facilitates the production of cyclotides labeled with NMR active isotopes, such as ^15^N and ^13^C, in a relatively inexpensive fashion [5]. This approach was used to carry out structural studies using heteronuclear NMR on a cyclotide engineered to bind the p53 binding domain of the E3-ligases Hdm2 and HdmX, allowing elucidation of the structure of the bioactive cyclotide bound to its target (Figure 3) [5].

## 6. Biological Activities of Naturally-occurring Cyclotides

Naturally-occurring cyclotides from the Möbius and bracelet sub-families seem to work mainly as host-defense agents, as deduced from their activities against insects [20,47,77,78,79,80]. Cyclotides from these two subfamilies have also been shown to inhibit the growth and development of nematodes and trematodes [81,82,83], and mollusks [84]; as well as antifungal activity shown by cyclotides in *Viola odorata* against the agriculturally-relevant, filamentous fungus *F. graminearum* [85].

Möbius and bracelet cyclotides interact with the cellular membranes of the gastrointestinal tract in insects, disrupting their normal function [86]. The molecular mechanism has been well studied for the Möbius cyclotide kalata B1, involving first the specific binding of the cyclotide to the phosphatidylethanolamine phospholipids present in the cellular membrane [87,88,89]. This compromises the membrane physical integrity, triggering the formation of pores and consequent leakage of the cellular contents [90,91,92,93].

Cyclotides from the Möbius and bracelet sub-families present some amphipathic characteristics as their molecular surfaces contain well-defined hydrophobic and hydrophilic patches [92]. This molecular feature resembles, to some extent, the amphipathic character of classical antimicrobial peptides. It is not surprising then that some cyclotides of this family show antibacterial activity [94]. Cyclotides kalata B1, and circulin A and B have shown minimal inhibitory concentrations (MICs) against *S. aureus* at 0.26 μM, 0.19 μM, 13.5 μM, and 39 μM, respectively, under low salt conditions, but no activity under physiological salt conditions [94].

Similar activities have also been found in other cyclotides isolated from *Hedyota biflora* (*Rubiaceae* family) [95,96] and *Clitoria ternatea* (*Fabaceae* family) [51]. The cyclotide with the highest antimicrobial activity tested so far is the bracelet cyclotide cycloviolacin O2, with reported MIC values of 8.75 µM, 2.2 µM, and >50 µM agaisnt *S. enterica*, *E. coli*, and *S. aureus*, respectively [97]. This cyclotide has showed antibacterial activity against the Gram-positive bacterium *Staphylococcus aureus* in a mouse infection model [98]. Intriguingly, the antimicrobial activity of cyclotides when tested in vitro strongly depends on the buffer composition occurring only under hypotonic conditions when low ionic strength buffers are used. This suggests that the antimicrobial activity observed in animal models may be due to an indirect effect of the cyclotide. The toxicity of some of the natural cyclotides tested so far shows that at the effective antimicrobial concentration they also show high toxicity against mammalian cells [99].

Several cyclotides have shown selective cytotoxicity against several cancer cell lines, including primary cancer cell lines, when compared to normal cells [100,101,102]. More recently, several cyclotides isolated from *Viola ignobilis* (*Violaceae* family), *Hedyotis* diffusa (*Rubiaceae* family), and *Pombalia calceolaria* have shown cytotoxicity against HeLa [103], several prostate cancer cell lines [104,105], and breast cancer, respectively [106]. Unfortunately, the therapeutic index (i.e., the ratio between the dose required for therapeutic effects versus toxic effects on normal cells) of cytotoxic cyclotides is not optimal yet, requiring optimization before that can be developed into effective anti-cancer therapeutic agents.

As mentioned earlier, the cyclotide kalata B1 was originally discovered as an efficient uterotonic agent used in a traditional medicine remedy to facilitate childbirth [35]. Recent studies on the related cyclotide kalata B7, which is also found in the same plant where kalata B1 was originally isolated from, have shown that this cyclotide is a moderate agonist of the G protein-coupled oxytocin and vasopressin V_1a_ receptors [107]. The activity of kalata B7, however, was modest (EC_50_ values ranging from 1 to 10 µM) when compared to that of the natural receptor ligands oxytocin and vasopressin (EC_50_ ≈ 1 nM) [107,108]. Cyclotide kalata B7, as with other kalata cyclotides (mainly kalata B1 and B2), also interacts strongly with phosphatidylethanolamine-containing lipids in cellular membranes causing its disruption [109]. This interaction is likely the cause of the hemolytic properties and cardiotoxicity observed in several cyclotides of the kalata B1 family, indicating that these types of cyclotides have to be further optimized before they can be used as potential therapeutic agents.

More recently, cyclotides isolated from an Ipecac root extract have been shown to antagonize the corticotropin releasing factor type 1 receptor (CRF1R) [110]. The most active cyclotide, caripe 8, was able to reduce corticotropin releasing factor (CRF) potency by ≈ 4.5-fold. In contrast, caripe 8 did not inhibit forskolin- or vasopressin-stimulated cAMP responses at the vasopressin V2 receptor, suggesting a CRF1R-specific mode-of-action.

Kalata B1 has been also shown to inhibit human prolyl oligopeptidase (POP) with an IC_50_ value of ≈ 6 µM. The inhibitory activity appeared to be selective for POP, since kalata B1 was not able to inhibit the proteolytic activity of trypsin or chymotrypsin [111]. The enzyme POP is well known for its role in memory and learning processes, and it is currently being considered as a promising therapeutic target for the cognitive deficits associated with several psychiatric and neurodegenerative diseases, such as schizophrenia and Parkinson’s disease. 

The cyclotide from *Viola odorata,* Cycloviolacin O2, has been shown to form pores in HIV infected T-cells and monocytes. In addition, it was also able to decrease the content of p24 in the HIV viral particle, disrupting the structural integrity of the virus. These effects could help the efficacy of classical retroviral therapeutic agents, although it has not been investigated yet [112]. 

Other cyclotides isolated from *O. affinis* have also anti-plasmodial activity against *Plasmodium berghei* as well as anti-inflammatory activity by decreasing the expression levels of pro-inflammatory mediators such as iNO and TNF-α [112].

## 7. Cyclotides with Novel Biological Activities

The unique properties associated with the cyclotide scaffold make it an excellent molecular framework for the design of a novel type of peptide-based therapeutics (see Table 1) [17,18,21,22]. Cyclotides are extremely resistant to physical, chemical, and biological degradation due to the highly rigid and compact structure provided by the CCK topology. Furthermore, the cyclotide scaffold is also highly tolerant to mutations and sequence insertions, making it an ideal molecular framework for production of novel cyclotides with new biological activities using molecular evolution and grafting techniques. Cyclotides from the trypsin inhibitor family are able to cross cellular membranes allowing them to target intracellular PPIs [5] and are not toxic to mammalian cells up to concentrations of 100 µM [5,28,29]. 

The potential to produce engineered cyclotides with novel biological activities was first reported in two reports aimed to the development of novel anti-viral [68] and anti-cancer peptide-based therapeutics [113,114]. Targeting angiogenesis is a validated molecular target for the development of anti-cancer therapeutics, as tumor growth requires neoangiogenesis. Molecular grafting of several Arg-rich polypeptide inhibitors of the vascular endothelial growth factor A (VEGF-A) into several loops of cyclotide kalata B1 produced several cyclotides with anti-VEGF activity [113]. The most active cyclotide inhibited the VEGF-A receptor with an IC_50_ value ≈ 12 µM. Although the biological activity would have to be improved by several orders of magnitude for in vivo testing, this early work showed the first example of a successful functional redesign of a naturally-occurring cyclotide by using an epitope or molecular grafting approach.

Similar molecular grafting approaches using the molecular framework of kalata B1 have been reported in the literature to target bradykinin and melanocortin 4 receptors for pain and obesity management, respectively [30,115]. The kalata B1-based bradykinin antagonist was the first engineered cyclotide to show activity when dosed orally [30]. A more recent study using a point mutated kalata B1 cyclotide also showed oral bioavailability in a mouse model of multiple sclerosis [31]. The treatment with this cyclotide impeded disease progression and did not exhibit adverse effects at a dose of 20 mg/kg [31]. Despite the fact that these two studies provided detailed pharmacokinetic or pharmacodynamic data on the corresponding engineered cyclotides, these studies highlight once more the potential of the cyclotide scaffold for the development of orally-bioavailable peptide-based therapeutics. 

Cyclotides from the trypsin inhibitory subfamily, mainly cyclotides MCoTI-I/II, have also been used as molecular templates to produce cyclotides with novel biological activities by employing molecular grafting (see Table 1). Cyclotide MCoTI-I has been successfully used for the design of potent antagonists for the cytokine receptor CXCR4 [116]. Many cancer cells show overexpression of the CXCR4 receptor, which is believed to drive tumor metastasis, neoangiogenesis, and tumor growth and survival [117]. CXCR4 cyclotide antagonists have also recently used bioimaging agents, showing the potential of cyclotides for this task for the first time [63]. This work used a [^64^Cu]-DOTA-labeled version of a CXCR4-binding MCoTI-based cyclotide for the efficient detection of tumors containing CXCR4-expressing cells in mice in combination with positron emission tomography-computed tomography (PET-CT) [63]. The results obtained in this study revealed high in vivo specificity and retention of the bioactive molecularly-targeted cyclotide, therefore also highlighting the potential of bioactive cyclotides for the development of new imaging agents that target CXCR4 [63].

Cyclotides from the trypsin inhibitor subfamily can also be used for the development of protease inhibitors with pharmacological relevance. Proteases are well-recognized drug targets, as they are involved many human diseases, including inflammatory diseases, cancer, cardiovascular, and neurodegenerative conditions [118,119]. For example, the introduction of different mutations onto loops 1 and 6 of cyclotide MCoTI-II converted it into a selective and potent foot-and-mouth-disease (FMDV) 3C protease inhibitor [68]. A similar approach was employed for the production of potent inhibitors against β-tryptase and human leukocyte elastase, which are validated targets for inflammatory disorders [114,120]. More recently, cyclotide MCoTI-II was also transformed into a highly potent kallikrein-related peptidase 4 (KLK4) inhibitor (*K*_i_ ≈ 0.1 nM) that displayed 100,000-fold selectivity over related KLKs. This was accomplished by grafting a preferred KLK4 cleavage sequence into loops 1 and 6 of cyclotide MCoTI-II [121]. The inhibitors were shown to be nontoxic to human cells and stable in human serum.

Cyclotide MCoTI-II was also used as a molecular scaffold to produce novel peptide-based inhibitors of the breakpoint cluster region protein-Abelson murine leukemia viral oncogene homolog (BCR-ABL) kinase [122]. In this case two peptide sequences derived from an optimal substrate for the Abl kinase were grafted into loops 1 or 6 of cyclotide MCoTI-II to produce several cyclotides able to show Abl kinase inhibition in vitro in the low micromolar range. However, these cyclotides did not show significant growth inhibition in an imatinib-sensitive human chronic myeloid leukemia (CML) cell line. Although this study did not investigate the inhibition of cytosolic BCR-ABL, the lack of activity suggests that more potent kinase inhibitors may be required to observe activity in cell and in vivo. Despite these results, this work represents an example of molecular grafting using two different loops to provide a novel cyclotide that was able to fold correctly and have moderate kinase inhibitory activity, which is a remarkable result.

MCoTI-grafted cyclotides have been also used to inhibit α-synuclein-induced cytotoxicity when expressed in baker’s yeast *Saccharomyces cerevisiae* [74]. The small lipid-binding protein α-synuclein has been linked to Parkinson’s disease by genetic evidence and abnormal presence in the Parkinson’s disease-associated intracellular aggregates, known as Lewy bodies [123], and therefore is a validated therapeutic target for Parkinson’s disease [124].

Among the unique features of the cyclotide scaffold, one of the most exciting is that some cyclotides can penetrate cells and access to cytosolic cellular fraction. This exceptional feature found in cyclotides from the trypsin inhibitor subfamily allows cyclotides to target intracellular PPIs. This was demonstrated on a report where the cyclotide MCoTI-I was engineered to produce a potent inhibitor for the interaction between p53 and the proteins Hdm2/HdmX (Figure 3) [5]. This MCoTI-based cyclotide was able to bind to the p53-binding domain of both Hdm2 and HdmX with very high affinity (*K*_d_ ≈ 2 and 10 nM for Hdm2 and HdmX, respectively), and showed high ex-vivo stability in serum. This cyclotide was cytotoxic to several wild-type p53 cancer cell lines by activating the p53 tumor suppressor pathway both in vitro and in vivo using an animal model of human colorectal carcinoma [5]. This work represents the first example of an engineered cyclotide able to target an intracellular PPI in vivo and shows the real therapeutic potential of MCoTI-based cyclotides for targeting intracellular PPIs in vivo. A similar approach but using cyclotide MCoTI-II instead was also reported to produce cyclotide antagonists of the Su(var)3-9/enhancer-of-zeste and Trithorax (SET) protein, which is overexpressed in some human cancers [125]. However, no in vivo results were reported in this study.

More recently, a point mutated cyclotide kalata B1 T20K was reported to have oral activity in a mouse model of multiple sclerosis [126]. This cyclotide has been recently shown to be able to modulate the activity of intracellular 14-3-3 proteins using chemical proteomic tools [127].

As mentioned earlier, in-cell production of natively folded cyclotides allows the production of large libraries of genetically-encoded cyclotides with complexities that could easily reach ≈ 10^9^, i.e., around a billion different cyclotide sequences. The production of such large libraries should allow the use of selections strategies mimicking the evolutionary processes that take place in nature for the selection of sequences that can bind specific molecular targets. The first example described the biosynthesis of a small library based on cyclotide MCoTI-I, where every residue in loops 1, 2, 3, 4, and 5 was mutated to evaluate the effects on folding and trypsin binding activity of the resulting mutants [128]. This early work produced a small library containing around 30 different cyclotides with single point mutations. Most of the mutations did not negatively affect the folding of the resulting cyclotides, emphasizing the high plasticity and sequence tolerance of MCoTI-based cyclotides to mutations [128].

An acyclic version of cyclotide kalata B1 has also been used for the generation of libraries in combination with a bacterial display approach that was later screened for selecting cyclotide sequences specific for the vascular endothelial growth factor A (VEGFA)binding site on neuropilin-1 [129]. Using this approach several cyclotides with high affinity (*K*_d_ ≈ 50 nM), high proteolytic resistance, and in vitro activity inhibiting endothelial cell migration (EC_50_ ≈100 nM) were reported [129]. 

A linearized version of cyclotide MCoTI-II was also employed as a scaffold to generate a genetically-encoded library that was screened using a yeast-display approach to select sequences able to bind to the cytotoxic T lymphocyte-associated antigen 4 (CTLA-4) [130]. CTLA-4 is an inhibitory receptor expressed by T lymphocytes that functions as an immune checkpoint downregulating immune responses, and has emerged as a target for the development of checkpoint inhibitors for cancer treatment [131].

A fully folded cyclotide-based genetically-encoded library was recently used for phenotypic in eukaryotic cells [74]. In this study, a bioactive cyclotide able to reduce α-synuclein-induced cytotoxicity in baker’s yeast *S. cerevisiae* was rapidly selected by phenotypic screening from cells transformed with a mixture of plasmids encoding active and inactive cyclotides at a ratio of 1 to 50,000 [74]. These results show the potential to perform rapid phenotypic screening of genetically encoded cyclotide-based libraries in eukaryotic cells for the selection of bioactive compounds using activity- rather than binding-based screening assays. Expression of cyclotide-based libraries using eukaryotic expression systems also allows the production of cyclotides with different post-translational modifications that are not available in bacterial expression systems in order to increase the molecular diversity of the library.

The availability of efficient methods for the chemical production of cyclotides has also made it possible to carry out high throughput screening on chemically-produced libraries of cyclotides [62]. A small amino acid scanning library based on a CXCR4 cyclotide antagonist was recently created by using a “tea-bag” approach in combination with a high efficiency “one-pot” cyclization protocol involving concomitant cyclization and oxidative folding [62]. This protocol also includes the use of an efficient purification step to rapidly remove the non-folded cyclotides from the cyclization-folding crude. This approach can be used for the purification of cyclotide or other disulfide-rich polypeptide mixtures, therefore making it possible to produce amino acid and positional scanning libraries to carry out efficient screening of large chemical-generated libraries. A similar approach was recently used for the production of potent inhibitors of the anthrax lethal factor protease (*K*_i_ ≈ 40 nM) and TNF-α converting enzyme (TACE) (*K*_i_ ≈ 150 nM) using the disulfide-rich backbone cyclized θ-defensin RTD-1 as a molecular scaffold [66].

## 8. Biodistribution Studies on Cyclotides

There are some published reports on the biodistribution and potential to cross the blood brain barrier of cyclotides from the trypsin inhibitor subfamily [63,132]. These two reports confirmed that naturally-occurring cyclotides MCoTI-I/II are distributed in mice predominantly into serum and kidneys, confirming high in vivo stability, and that they are eliminated mostly through renal clearance [63,132]. In addition, it was also confirmed that cyclotide MCoTI-II cannot cross the blood brain barrier [132]. It is important to notice, however, that the biodistribution profile of cyclotides will also depend strongly on their biological activity. For example, a study on the biodistribution of a CXCR4 binding cyclotide showed major accumulation in the lungs, liver, and spleen, even after 24 h of administration [63]. This cyclotide was also mostly secreted though renal clearance, showing a peak after 90 min of administration and slowly decaying after 24 h. Similar studies have also been carried out to study the pharmacokinetic profile of cyclotide kalata B1 in rats, obtaining very similar results to those found for MCoTI-cylcotide [133].

Unfortunately, there is not much data published on the pharmacokinetic or pharmacodynamic profiles of bioactive cyclotides, which is surprising given the good in vivo biological activity of some engineered cyclotides. 

## 9. Summary

It is becoming clear that cyclotides are now a well-studied family of micro-proteins that are starting to gain acceptance as molecular scaffolds for the potential design of novel peptide-based therapeutics and diagnostic tools based on their unique properties. Cyclotides possess a unique highly constrained structure containing a cystine-knot and circular backbone topology, which confers them an extraordinary stability to thermal and chemical denaturation, and proteolytic degradation. Some cyclotides can be dosed orally and can cross cellular membranes, allowing them to target extracellular, and more importantly intracellular, PPIs in vivo [5,30,31,127]. This highlights the high stability of the circular cystine knot topology to degradation-reduction under complex biological conditions. Cyclotides are relatively small polypeptides that can be readily accessed using standard solid-phase peptide synthesis methods, allowing the introduction of chemical modifications, such as non-natural amino acids and PEGylation, to improve their pharmacological profiles [64,116]. More importantly, naturally occurring cyclotides are also able to tolerate substantial sequence variation and have been produced using several heterologous systems, making them ideal substrates for molecular evolution for the selection of novel sequences with optimal binding and inhibitory characteristics against specific molecular targets [74,129,130]. Large scale production of bioactive cyclotides can easily be accomplished by using heterologous expression systems. The full characterization of AEP-like ligases involved in the biosynthesis of cyclotides should allow the production of genetically-modified plants able to biosynthesize cyclotides. In addition, the use of PTS-mediated backbone cyclization employing microbial expression systems, such as the bacterium *E. coli*, provides expression yields of cyclotides that are comparable to those found in plants when grown in vitro. The simplicity of growing bacteria, as well as their significantly faster growth rates, makes them an attractive alternative to the low-cost production of bioactive cyclotides at large scale. All these unique properties make cyclotides one of the most promising scaffolds available now for the design of novel peptide-based therapeutics.

## 10. Concluding Remarks

The selective and effective disruption PPIs still remains a difficult task. This is mostly due to the nature of the interacting surfaces, which are usually large and relatively flat. The cyclotide scaffold provides a cell-permeable and highly-constrained molecular framework to efficiently target both extra- and intracellular PPIs. There are proven tools available to produce cyclotides with novel biological activities by employing molecular grafting of bioactive epitopes, or even through molecular evolution techniques. Using these tools, cyclotides that can target a multitude of protein targets (see Table 1) have been designed and tested mostly in vitro, although some in animal models also [5,30,31]. A few examples have also shown the potential of cyclotides to be dosed orally to target specific molecular targets or diseases [30,31], although this property would need to be tested in any new cyclotide, as changes in sequence could have a negative effect on this interesting property.

Despite this initial success in employing the cyclotide scaffold to target specific proteins to modulate their biological activity, no cyclotides have reached human clinical trials yet. Among the different challenges that affect bioactive cyclotides before they can move into the clinic are the potential immunogenicity and oral bioavailability. 

Although cyclotides are highly constrained and extremely resistant to proteolytic cleavage, which is required for proper T-epitope presentation and activation of the cellular immune response, more detailed studies on the immunogenicity of bioactive cyclotides are required. In this regard, a recent study was able to rise polyclonal antibodies against cyclotide cycloviolacin O2, however due to the poor immunogenicity of the free cyclotide conjugation to an immunogenic protein, a carrier was required [140]. 

As indicated earlier, some cyclotides have been proven to be orally active, however no detailed information has been released yet on their pharmacological profiles when dosed orally. It is anticipated, however, that more studies on the biopharmaceutical properties of these exciting new micro-proteins may be available very soon. 

## Figures and Tables

**Table 1:** Engineered cyclotides described in the literature with novel biological activities leading to bioimaging and therapeutic applications. Table adapted and updated from a previous study [17].

Cyclotide	Biological Activity	Loop Modified	Application	Ref.
**Möbius Subfamily**				
Kalata B1	VEGF-A antagonist	2, 3, 5, and 6	Anti-angiogenic, potential anti-cancer activity	[113]
Kalata B1	Dengue NS2B-NS3 Protease inhibitor	2 and 5	Anti-viral for Dengue virus infections	[134]
Kalata B1	Bradikynin B1 receptor antagonist	6	Chronic and inflammatory pain	[30]
Kalata B1	Melanocortin 4 receptor Agonist	6	Obesity	[115]
Kalata B1	Neuropilin-1/2 antagonist	5 and 6	Inhibition of endothelial cell migration and angiogenesis	[129]
Kalata B1	Immunomodulator	5 and 6	Protecting against multiple sclerosis	[135]
Kalata B1	Immunomodulator	4	Protecting against multiple sclerosis	[31]
**Trypsin Inhibitor Subfamily**				
MCoTI-I	CXCR4 antagonist	6	Anti-metastatic and anti-HIV PET-CT imaging	[62,63,116]
MCoTI-I	p53-Hdm2/HdmX	6	Anti-tumor by activation of p53 pathway	[5]
MCoTI-II	FMDV 3C protease Inhibitor	1	Anti-viral for foot-and-mouth disease	[68]
MCoTI-II	β-Tryptase inhibitor	3, 5, and 6	Inflammation diseases	[120]
MCoTI-II	β-Tryptase inhibitor Human elastase inhibitor	1	Inflammation diseases	[114]
MCoTI-II	CTLA-4 antagonist	1,3, and 6	Immunotherapy for cancer	[130]
MCoTI-II	Tryptase inhibitor	1	Anti-cancer	[43]
MCoTI-II	VEGF receptor agonist	6	Wound healing and cardiovascular damage	[136]
MCoTI-I	α-Synuclein-induced cytotoxicity inhibitor	6	Parkinson’s disease Validate phenotypic screening of genetically-encoded cyclotide libraries	[74]
MCoTI-II	BCR-Abl kinase Inhibitor	1 and 6	Chronic myeloid leukemia Attempt to graft both a cell penetrating peptide and kinase inhibitor	[122]
MCoTI-I	MAS1 receptor agonist	6	Lung cancer and myocardial infarction	[64]
MCoTI-II	SET antagonist	6	Potential anticancer	[125]
MCoTI-II	FXIIa and FXa inhibitors	1 and 6	Antithrombotic and cardiovascular disease	[137]
MCoTI-II	Thrombospondin-1 (TSP-1) agonist	6	Microvascular endothelial cell migration inhibition Anti-angiogenesis	[138]
MCoTI-II	Antiangiogenic	5 and 6	Anti-cancer	[139]
MCoTI-II	Kallikrein 4 (KLK4) inhibitor	1 and 8	Anti-cancer	[121]

## References

[1] Jubb H., Higueruelo A.P., Winter A., Blundell T.L. (2012). Structural biology and drug discovery for protein-protein interactions. Trends Pharmacol. Sci..

[2] Zinzalla G., Thurston D.E. (2009). Targeting protein-protein interactions for therapeutic intervention: A challenge for the future. Future Med. Chem..

[3] Fry D.C., Vassilev L.T. (2005). Targeting protein-protein interactions for cancer therapy. J. Mol. Med..

[4] Qian Z., Dougherty P.G., Pei D. (2017). Targeting intracellular protein-protein interactions with cell-permeable cyclic peptides. Curr. Opin. Chem. Biol..

[5] Ji Y., Majumder S., Millard M., Borra R., Bi T., Elnagar A.Y., Neamati N., Shekhtman A., Camarero J.A. (2013). In vivo activation of the p53 tumor suppressor pathway by an engineered cyclotide. J. Am. Chem. Soc..

[6] Sheng C., Dong G., Miao Z., Zhang W., Wang W. (2015). State-of-the-art strategies for targeting protein–protein interactions by small-molecule inhibitors. Chem. Soc. Rev..

[7] Laraia L., McKenzie G., Spring D.R., Venkitaraman A.R., Huggins D.J. (2015). Overcoming chemical, biological, and computational challenges in the development of inhibitors targeting protein-protein interactions. Chem. Biol..

[8] Stumpp M.T., Binz H.K., Amstutz P. (2008). Darpins: A new generation of protein therapeutics. Drug Discov. Today.

[9] Ferrara N., Hillan K.J., Gerber H.P., Novotny W. (2004). Discovery and development of bevacizumab, an anti-vegf antibody for treating cancer. Nat. Rev. Drug Discov..

[10] Holliger P., Hudson P.J. (2005). Engineered antibody fragments and the rise of single domains. Nat. Biotechnol..

[11] Klint J.K., Senff S., Rupasinghe D.B., Er S.Y., Herzig V., Nicholson G.M., King G.F. (2012). Spider-venom peptides that target voltage-gated sodium channels: Pharmacological tools and potential therapeutic leads. Toxicon.

[12] Wurch T., Pierre A., Depil S. (2012). Novel protein scaffolds as emerging therapeutic proteins: From discovery to clinical proof-of-concept. Trends Biotechnol..

[13] Lewis R.J. (2012). Discovery and development of the chi-conopeptide class of analgesic peptides. Toxicon.

[14] Sancheti H., Camarero J.A. (2009). “Splicing up” drug discovery. Cell-based expression and screening of genetically-encoded libraries of backbone-cyclized polypeptides. Adv. Drug Deliv. Rev..

[15] Bloom L., Calabro V. (2009). Fn3: A new protein scaffold reaches the clinic. Drug Discov. Today.

[16] Lewis R.J. (2009). Conotoxin venom peptide therapeutics. Adv. Exp. Med. Biol..

[17] Chaudhuri D., Aboye T., Camarero J.A. (2019). Using backbone-cyclized cys-rich polypeptides as molecular scaffolds to target protein-protein interactions. Biochem. J.

[18] Wang C.K., Craik D.J. (2018). Designing macrocyclic disulfide-rich peptides for biotechnological applications. Nat. Chem. Biol..

[19] Craik D.J., Lee M.H., Rehm F.B.H., Tombling B., Doffek B., Peacock H. (2018). Ribosomally-synthesised cyclic peptides from plants as drug leads and pharmaceutical scaffolds. Biorg. Med. Chem..

[20] Poth A.G., Colgrave M.L., Lyons R.E., Daly N.L., Craik D.J. (2011). Discovery of an unusual biosynthetic origin for circular proteins in legumes. Proc. Natl. Acad. Sci. USA.

[21] Gould A., Camarero J.A. (2017). Cyclotides: Overview and biotechnological applications. ChemBioChem.

[22] Craik D.J., Du J. (2017). Cyclotides as drug design scaffolds. Curr. Opin. Chem. Biol..

[23] Camarero J.A. (2017). Cyclotides, a versatile ultrastable micro-protein scaffold for biotechnological applications. Bioorg. Med. Chem. Lett..

[24] Rosengren K.J., Daly N.L., Plan M.R., Waine C., Craik D.J. (2003). Twists, knots, and rings in proteins. Structural definition of the cyclotide framework. J. Biol. Chem..

[25] Felizmenio-Quimio M.E., Daly N.L., Craik D.J. (2001). Circular proteins in plants: Solution structure of a novel macrocyclic trypsin inhibitor from momordica cochinchinensis. J. Biol. Chem..

[26] Saether O., Craik D.J., Campbell I.D., Sletten K., Juul J., Norman D.G. (1995). Elucidation of the primary and three-dimensional structure of the uterotonic polypeptide kalata b1. Biochemistry.

[27] Li Y., Bi T., Camarero J.A. (2015). Chemical and biological production of cyclotides. Adv. Bot. Res..

[28] Contreras J., Elnagar A.Y., Hamm-Alvarez S.F., Camarero J.A. (2011). Cellular uptake of cyclotide mcoti-i follows multiple endocytic pathways. J. Control. Release.

[29] Cascales L., Henriques S.T., Kerr M.C., Huang Y.H., Sweet M.J., Daly N.L., Craik D.J. (2011). Identification and characterization of a new family of cell-penetrating peptides: Cyclic cell-penetrating peptides. J. Biol. Chem..

[30] Wong C.T., Rowlands D.K., Wong C.H., Lo T.W., Nguyen G.K., Li H.Y., Tam J.P. (2012). Orally active peptidic bradykinin b1 receptor antagonists engineered from a cyclotide scaffold for inflammatory pain treatment. Angew. Chem. Int. Ed. Engl..

[31] Thell K., Hellinger R., Sahin E., Michenthaler P., Gold-Binder M., Haider T., Kuttke M., Liutkeviciute Z., Goransson U., Grundemann C. (2016). Oral activity of a nature-derived cyclic peptide for the treatment of multiple sclerosis. Proc. Natl. Acad. Sci. USA.

[32] Puttamadappa S.S., Jagadish K., Shekhtman A., Camarero J.A. (2010). Backbone dynamics of cyclotide mcoti-i free and complexed with trypsin. Angew. Chem. Int. Ed. Engl..

[33] Colgrave M.L., Craik D.J. (2004). Thermal, chemical, and enzymatic stability of the cyclotide kalata b1: The importance of the cyclic cystine knot. Biochemistry.

[34] Garcia A.E., Camarero J.A. (2010). Biological activities of natural and engineered cyclotides, a novel molecular scaffold for peptide-based therapeutics. Curr. Mol. Pharmacol..

[35] Gran L. (1973). Oxytocic principles of oldenlandia affinis. Lloydia.

[36] Gran L. (1973). On the effect of a polypeptide isolated from “kalata-kalata” (oldenlandia affinis dc) on the oestrogen dominated uterus. Acta Pharmacol. Toxicol..

[37] Weidmann J., Craik D.J. (2016). Discovery, structure, function, and applications of cyclotides: Circular proteins from plants. J. Exp. Bot..

[38] Wang C.K., Kaas Q., Chiche L., Craik D.J. (2008). Cybase: A database of cyclic protein sequences and structures, with applications in protein discovery and engineering. Nucleic Acids Res..

[39] Aboye T.L., Clark R.J., Burman R., Roig M.B., Craik D.J., Goransson U. (2011). Interlocking disulfides in circular proteins: Toward efficient oxidative folding of cyclotides. Antioxid. Redox Signal..

[40] Heitz A., Hernandez J.F., Gagnon J., Hong T.T., Pham T.T., Nguyen T.M., Le-Nguyen D., Chiche L. (2001). Solution structure of the squash trypsin inhibitor mcoti-ii. A new family for cyclic knottins. Biochemistry.

[41] Mylne J.S., Chan L.Y., Chanson A.H., Daly N.L., Schaefer H., Bailey T.L., Nguyencong P., Cascales L., Craik D.J. (2012). Cyclic peptides arising by evolutionary parallelism via asparaginyl-endopeptidase-mediated biosynthesis. Plant Cell.

[42] Du J., Chan L.Y., Poth A.G., Craik D.J. (2019). Discovery and characterization of cyclic and acyclic trypsin inhibitors from momordica dioica. J. Nat. Prod..

[43] Quimbar P., Malik U., Sommerhoff C.P., Kaas Q., Chan L.Y., Huang Y.H., Grundhuber M., Dunse K., Craik D.J., Anderson M.A. (2013). High-affinity cyclic peptide matriptase inhibitors. J. Biol. Chem..

[44] Chiche L., Heitz A., Gelly J.C., Gracy J., Chau P.T., Ha P.T., Hernandez J.F., Le-Nguyen D. (2004). Squash inhibitors: From structural motifs to macrocyclic knottins. Curr. Protein Pept. Sci..

[45] Ravipati A.S., Henriques S.T., Poth A.G., Kaas Q., Wang C.K., Colgrave M.L., Craik D.J. (2015). Lysine-rich cyclotides: A new subclass of circular knotted proteins from violaceae. ACS Chem. Biol..

[46] Craik D.J., Malik U. (2013). Cyclotide biosynthesis. Curr. Opin. Chem. Biol..

[47] Jennings C., West J., Waine C., Craik D., Anderson M. (2001). Biosynthesis and insecticidal properties of plant cyclotides: The cyclic knotted proteins from oldenlandia affinis. Proc. Natl. Acad. Sci. USA.

[48] Arnison P.G., Bibb M.J., Bierbaum G., Bowers A.A., Bugni T.S., Bulaj G., Camarero J.A., Campopiano D.J., Challis G.L., Clardy J. (2013). Ribosomally synthesized and post-translationally modified peptide natural products: Overview and recommendations for a universal nomenclature. Nat. Prod. Rep..

[49] Saska I., Gillon A.D., Hatsugai N., Dietzgen R.G., Hara-Nishimura I., Anderson M.A., Craik D.J. (2007). An asparaginyl endopeptidase mediates in vivo protein backbone cyclization. J. Biol. Chem..

[50] Poth A.G., Mylne J.S., Grassl J., Lyons R.E., Millar A.H., Colgrave M.L., Craik D.J. (2012). Cyclotides associate with leaf vasculature and are the products of a novel precursor in petunia (solanaceae). J. Biol. Chem..

[51] Nguyen G.K., Zhang S., Nguyen N.T., Nguyen P.Q., Chiu M.S., Hardjojo A., Tam J.P. (2011). Discovery and characterization of novel cyclotides originated from chimeric precursors consisting of albumin-1 chain a and cyclotide domains in the fabaceae family. J. Biol. Chem..

[52] Gillon A.D., Saska I., Jennings C.V., Guarino R.F., Craik D.J., Anderson M.A. (2008). Biosynthesis of circular proteins in plants. Plant J..

[53] Nguyen G.K., Wang S., Qiu Y., Hemu X., Lian Y., Tam J.P. (2014). Butelase 1 is an asx-specific ligase enabling peptide macrocyclization and synthesis. Nat. Chem. Biol..

[54] Harris K.S., Durek T., Kaas Q., Poth A.G., Gilding E.K., Conlan B.F., Saska I., Daly N.L., van der Weerden N.L., Craik D.J. (2015). Efficient backbone cyclization of linear peptides by a recombinant asparaginyl endopeptidase. Nat. Commun..

[55] Poon S., Harris K.S., Jackson M.A., McCorkelle O.C., Gilding E.K., Durek T., van der Weerden N.L., Craik D.J., Anderson M.A. (2018). Co-expression of a cyclizing asparaginyl endopeptidase enables efficient production of cyclic peptides in planta. J. Exp. Bot..

[56] Bernath-Levin K., Nelson C., Elliott A.G., Jayasena A.S., Millar A.H., Craik D.J., Mylne J.S. (2015). Peptide macrocyclization by a bifunctional endoprotease. Chem. Biol..

[57] Hemu X., Qiu Y., Nguyen G.K., Tam J.P. (2016). Total synthesis of circular bacteriocins by butelase 1. J. Am. Chem. Soc..

[58] Nguyen G.K., Hemu X., Quek J.P., Tam J.P. (2016). Butelase-mediated macrocyclization of d-amino-acid-containing peptides. Angew. Chem. Int. Ed. Engl..

[59] Nguyen G.K., Qiu Y., Cao Y., Hemu X., Liu C.F., Tam J.P. (2016). Butelase-mediated cyclization and ligation of peptides and proteins. Nat. Protoc..

[60] Jackson M.A., Gilding E.K., Shafee T., Harris K.S., Kaas Q., Poon S., Yap K., Jia H., Guarino R., Chan L.Y. (2018). Molecular basis for the production of cyclic peptides by plant asparaginyl endopeptidases. Nat. Commun..

[61] Zauner F.B., Elsasser B., Dall E., Cabrele C., Brandstetter H. (2018). Structural analyses of arabidopsis thaliana legumain gamma reveal differential recognition and processing of proteolysis and ligation substrates. J. Biol. Chem..

[62] Aboye T., Kuang Y., Neamati N., Camarero J.A. (2015). Rapid parallel synthesis of bioactive folded cyclotides by using a tea-bag approach. ChemBioChem.

[63] Lesniak W.G., Aboye T., Chatterjee S., Camarero J.A., Nimmagadda S. (2017). In vivo evaluation of an engineered cyclotide as specific cxcr4 imaging reagent. Chemistry.

[64] Aboye T., Meeks C.J., Majumder S., Shekhtman A., Rodgers K., Camarero J.A. (2016). Design of a mcoti-based cyclotide with angiotensin (1-7)-like activity. Molecules.

[65] Aboye T.L., Li Y., Majumder S., Hao J., Shekhtman A., Camarero J.A. (2012). Efficient one-pot cyclization/folding of rhesus theta-defensin-1 (rtd-1). Bioorg. Med. Chem. Lett..

[66] Li Y., Gould A., Aboye T., Bi T., Breindel L., Shekhtman A., Camarero J.A. (2017). Full sequence amino acid scanning of theta-defensin rtd-1 yields a potent anthrax lethal factor protease inhibitor. J. Med. Chem..

[67] Yang R., Wong Y.H., Nguyen G.K.T., Tam J.P., Lescar J., Wu B. (2017). Engineering a catalytically efficient recombinant protein ligase. J. Am. Chem. Soc..

[68] Thongyoo P., Roque-Rosell N., Leatherbarrow R.J., Tate E.W. (2008). Chemical and biomimetic total syntheses of natural and engineered mcoti cyclotides. Org. Biomol. Chem..

[69] Jia X., Kwon S., Wang C.I., Huang Y.H., Chan L.Y., Tan C.C., Rosengren K.J., Mulvenna J.P., Schroeder C.I., Craik D.J. (2014). Semienzymatic cyclization of disulfide-rich peptides using sortase a. J. Biol. Chem..

[70] Aboye T.L., Camarero J.A. (2012). Biological synthesis of circular polypeptides. J. Biol. Chem..

[71] Kimura R.H., Tran A.T., Camarero J.A. (2006). Biosynthesis of the cyclotide kalata b1 by using protein splicing. Angew. Chem. Int. Ed. Engl..

[72] Austin J., Kimura R.H., Woo Y.H., Camarero J.A. (2010). In vivo biosynthesis of an ala-scan library based on the cyclic peptide sfti-1. Amino Acids.

[73] Jagadish K., Borra R., Lacey V., Majumder S., Shekhtman A., Wang L., Camarero J.A. (2013). Expression of fluorescent cyclotides using protein trans-splicing for easy monitoring of cyclotide-protein interactions. Angew. Chem. Int. Ed. Engl..

[74] Jagadish K., Gould A., Borra R., Majumder S., Mushtaq Z., Shekhtman A., Camarero J.A. (2015). Recombinant expression and phenotypic screening of a bioactive cyclotide against alpha-synuclein-induced cytotoxicity in baker’s yeast. Angew. Chem. Int. Ed. Engl..

[75] Jagadish K., Camarero J.A. (2017). Recombinant expression of cyclotides using split inteins. Methods Mol. Biol..

[76] Seydel P., Dornenburg H. (2006). Establishment of in vitro plants, cell and tissue cultures from oldenlandia affinis for the production of cyclic peptides. Plant Cell Tissue Organ Cult..

[77] Jennings C.V., Rosengren K.J., Daly N.L., Plan M., Stevens J., Scanlon M.J., Waine C., Norman D.G., Anderson M.A., Craik D.J. (2005). Isolation, solution structure, and insecticidal activity of kalata b2, a circular protein with a twist: Do mobius strips exist in nature?. Biochemistry.

[78] Pinto M.F., Fensterseifer I.C., Migliolo L., Sousa D.A., de Capdville G., Arboleda-Valencia J.W., Colgrave M.L., Craik D.J., Magalhaes B.S., Dias S.C. (2012). Identification and structural characterization of novel cyclotide with activity against an insect pest of sugar cane. J. Biol. Chem..

[79] Craik D.J. (2012). Host-defense activities of cyclotides. Toxins.

[80] Gilding E.K., Jackson M.A., Poth A.G., Henriques S.T., Prentis P.J., Mahatmanto T., Craik D.J. (2016). Gene coevolution and regulation lock cyclic plant defence peptides to their targets. New Phytol..

[81] Colgrave M.L., Kotze A.C., Huang Y.H., O’Grady J., Simonsen S.M., Craik D.J. (2008). Cyclotides: Natural, circular plant peptides that possess significant activity against gastrointestinal nematode parasites of sheep. Biochemistry.

[82] Colgrave M.L., Kotze A.C., Ireland D.C., Wang C.K., Craik D.J. (2008). The anthelmintic activity of the cyclotides: Natural variants with enhanced activity. ChemBioChem.

[83] Malagon D., Botterill B., Gray D.J., Lovas E., Duke M., Gray C., Kopp S.R., Knott L.M., McManus D.P., Daly N.L. (2013). Anthelminthic activity of the cyclotides (kalata b1 and b2) against schistosome parasites. Biopolymers.

[84] Plan M.R., Saska I., Cagauan A.G., Craik D.J. (2008). Backbone cyclised peptides from plants show molluscicidal activity against the rice pest pomacea canaliculata (golden apple snail). J. Agric. Food Chem..

[85] Parsley N.C., Kirkpatrick C.L., Crittenden C.M., Rad J.G., Hoskin D.W. (2018). Pepsavi-ms reveals anticancer and antifungal cycloviolacins in viola odorata. Phytochemistry.

[86] Barbeta B.L., Marshall A.T., Gillon A.D., Craik D.J., Anderson M.A. (2008). Plant cyclotides disrupt epithelial cells in the midgut of lepidopteran larvae. Proc. Natl. Acad. Sci. USA.

[87] Troeira Henriques S., Huang Y.H., Chaousis S., Wang C.K., Craik D.J. (2014). Anticancer and toxic properties of cyclotides are dependent on phosphatidylethanolamine phospholipid targeting. ChemBioChem.

[88] Henriques S.T., Craik D.J. (2012). Importance of the cell membrane on the mechanism of action of cyclotides. ACS Chem. Biol..

[89] Henriques S.T., Huang Y.H., Castanho M.A., Bagatolli L.A., Sonza S., Tachedjian G., Daly N.L., Craik D.J. (2012). Phosphatidylethanolamine binding is a conserved feature of cyclotide-membrane interactions. J. Biol. Chem..

[90] Huang Y.H., Colgrave M.L., Daly N.L., Keleshian A., Martinac B., Craik D.J. (2009). The biological activity of the prototypic cyclotide kalata b1 is modulated by the formation of multimeric pores. J. Biol. Chem..

[91] Henriques S.T., Huang Y.H., Rosengren K.J., Franquelim H.G., Carvalho F.A., Johnson A., Sonza S., Tachedjian G., Castanho M.A., Daly N.L. (2011). Decoding the membrane activity of the cyclotide kalata b1: The importance of phosphatidylethanolamine phospholipids and lipid organization on hemolytic and anti-hiv activities. J. Biol. Chem..

[92] Troeira Henriques S., Craik D.J. (2017). Cyclotide structure and function: The role of membrane binding and permeation. Biochemistry.

[93] Cranfield C.G., Henriques S.T., Martinac B., Duckworth P., Craik D.J., Cornell B. (2017). Kalata b1 and kalata b2 have a surfactant-like activity in phosphatidylethanolomine-containing lipid membranes. Langmuir.

[94] Stromstedt A.A., Park S., Burman R., Goransson U. (2017). Bactericidal activity of cyclotides where phosphatidylethanolamine-lipid selectivity determines antimicrobial spectra. Biochimica Et Biophysica Acta-Biomembranes.

[95] Nguyen G.K., Zhang S., Wang W., Wong C.T., Nguyen N.T., Tam J.P. (2011). Discovery of a linear cyclotide from the bracelet subfamily and its disulfide mapping by top-down mass spectrometry. J. Biol. Chem..

[96] Wong C.T., Taichi M., Nishio H., Nishiuchi Y., Tam J.P. (2011). Optimal oxidative folding of the novel antimicrobial cyclotide from hedyotis biflora requires high alcohol concentrations. Biochemistry.

[97] Pranting M., Loov C., Burman R., Goransson U., Andersson D.I. (2010). The cyclotide cycloviolacin o2 from viola odorata has potent bactericidal activity against gram-negative bacteria. J. Antimicrob. Chemother..

[98] Fensterseifer I.C., Silva O.N., Malik U., Ravipati A.S., Novaes N.R., Miranda P.R., Rodrigues E.A., Moreno S.E., Craik D.J., Franco O.L. (2015). Effects of cyclotides against cutaneous infections caused by staphylococcus aureus. Peptides.

[99] He W., Chan L.Y., Zeng G., Daly N.L., Craik D.J., Tana N. (2011). Isolation and characterization of cytotoxic cyclotides from viola philippica. Peptides.

[100] Lindholm P., Goransson U., Johansson S., Claeson P., Gullbo J., Larsson R., Bohlin L., Backlund A. (2002). Cyclotides: A novel type of cytotoxic agents. Mol. Cancer Ther..

[101] Svangard E., Goransson U., Hocaoglu Z., Gullbo J., Larsson R., Claeson P., Bohlin L. (2004). Cytotoxic cyclotides from viola tricolor. J. Nat. Prod..

[102] Herrmann A., Burman R., Mylne J.S., Karlsson G., Gullbo J., Craik D.J., Clark R.J., Goransson U. (2008). The alpine violet, viola biflora, is a rich source of cyclotides with potent cytotoxicity. Phytochemistry.

[103] Esmaeili M.A., Abagheri-Mahabadi N., Hashempour H., Farhadpour M., Gruber C.W., Ghassempour A. (2016). Viola plant cyclotide vigno 5 induces mitochondria-mediated apoptosis via cytochrome c release and caspases activation in cervical cancer cells. Fitoterapia.

[104] Hu E., Wang D., Chen J., Tao X. (2015). Novel cyclotides from hedyotis diffusa induce apoptosis and inhibit proliferation and migration of prostate cancer cells. Int. J. Clin. Exp. Med..

[105] Zhang H., Song T., Yang Y., Fu C., Li J. (2018). Exploring the interaction mechanism between cyclopeptide dc3 and androgen receptor using molecular dynamics simulations and free energy calculations. Front. Chem..

[106] Pinto M.E., Najas J.Z., Magalhães L.G., Bobey A.F., Mendonça J.N., Lopes N.P., Leme F.M., Teixeira S.P., Trovó M., Andricopulo A.D. (2018). Inhibition of breast cancer cell migration by cyclotides isolated from pombalia calceolaria. J. Nat. Prod..

[107] Koehbach J., O’Brien M., Muttenthaler M., Miazzo M., Akcan M., Elliott A.G., Daly N.L., Harvey P.J., Arrowsmith S., Gunasekera S. (2013). Oxytocic plant cyclotides as templates for peptide g protein-coupled receptor ligand design. Proc. Natl. Acad. Sci. USA.

[108] Keov P., Liutkeviciute Z., Hellinger R., Clark R.J., Gruber C.W. (2018). Discovery of peptide probes to modulate oxytocin-type receptors of insects. Sci. Rep..

[109] Stromstedt A.A., Kristiansen P.E., Gunasekera S., Grob N., Skjeldal L., Goransson U. (2016). Selective membrane disruption by the cyclotide kalata b7: Complex ions and essential functional groups in the phosphatidylethanolamine binding pocket. Biochim. Biophys. Acta.

[110] Fahradpour M., Keov P., Tognola C., Perez-Santamarina E., McCormick P.J., Ghassempour A., Gruber C.W. (2017). Cyclotides isolated from an ipecac root extract antagonize the corticotropin releasing factor type 1 receptor. Front. Pharmacol..

[111] Hellinger R., Koehbach J., Puigpinos A., Clark R.J., Tarrago T., Giralt E., Gruber C.W. (2015). Inhibition of human prolyl oligopeptidase activity by the cyclotide psysol 2 isolated from psychotria solitudinum. J. Nat. Prod..

[112] Nworu C.S., Ejikeme T.I., Ezike A.C., Ndu O., Akunne T.C., Onyeto C.A., Okpalanduka P., Akah P.A. (2017). Anti-plasmodial and anti-inflammatory activities of cyclotide-rich extract and fraction of oldenlandia affinis (R. & S.) D.C. (rubiaceae). Afr. Health Sci..

[113] Gunasekera S., Foley F.M., Clark R.J., Sando L., Fabri L.J., Craik D.J., Daly N.L. (2008). Engineering stabilized vascular endothelial growth factor-a antagonists: Synthesis, structural characterization, and bioactivity of grafted analogues of cyclotides. J. Med. Chem..

[114] Thongyoo P., Bonomelli C., Leatherbarrow R.J., Tate E.W. (2009). Potent inhibitors of beta-tryptase and human leukocyte elastase based on the mcoti-ii scaffold. J. Med. Chem..

[115] Eliasen R., Daly N.L., Wulff B.S., Andresen T.L., Conde-Frieboes K.W., Craik D.J. (2012). Design, synthesis, structural and functional characterization of novel melanocortin agonists based on the cyclotide kalata b1. J. Biol. Chem..

[116] Aboye T.L., Ha H., Majumder S., Christ F., Debyser Z., Shekhtman A., Neamati N., Camarero J.A. (2012). Design of a novel cyclotide-based cxcr4 antagonist with anti-human immunodeficiency virus (hiv)-1 activity. J. Med. Chem..

[117] Balkwill F. (2004). The significance of cancer cell expression of the chemokine receptor cxcr4. Semin. Cancer Biol..

[118] Culp E., Wright G.D. (2017). Bacterial proteases, untapped antimicrobial drug targets. J. Antibiot..

[119] Gialeli C., Theocharis A.D., Karamanos N.K. (2011). Roles of matrix metalloproteinases in cancer progression and their pharmacological targeting. FEBS J..

[120] Sommerhoff C.P., Avrutina O., Schmoldt H.U., Gabrijelcic-Geiger D., Diederichsen U., Kolmar H. (2010). Engineered cystine knot miniproteins as potent inhibitors of human mast cell tryptase beta. J. Mol. Biol..

[121] Swedberg J.E., Ghani H.A., Harris J.M., de Veer S.J., Craik D.J. (2018). Potent, selective, and cell-penetrating inhibitors of kallikrein-related peptidase 4 based on the cyclic peptide mcoti-ii. ACS Med. Chem. Lett..

[122] Huang Y.H., Henriques S.T., Wang C.K., Thorstholm L., Daly N.L., Kaas Q., Craik D.J. (2015). Design of substrate-based bcr-abl kinase inhibitors using the cyclotide scaffold. Sci. Rep..

[123] Abeliovich A., Gitler A.D. (2016). Defects in trafficking bridge parkinson’s disease pathology and genetics. Nature.

[124] Zeuner K.E., Schaffer E., Hopfner F., Bruggemann N., Berg D. (2019). Progress of pharmacological approaches in parkinson’s disease. Clin. Pharmacol. Ther..

[125] D’Souza C., Henriques S.T., Wang C.K., Cheneval O., Chan L.Y., Bokil N.J., Sweet M.J., Craik D.J. (2016). Using the mcoti-ii cyclotide scaffold to design a stable cyclic peptide antagonist of set, a protein overexpressed in human cancer. Biochemistry.

[126] Thell K., Hellinger R., Schabbauer G., Gruber C.W. (2014). Immunosuppressive peptides and their therapeutic applications. Drug Discov. Today.

[127] Hellinger R., Thell K., Vasileva M., Muhammad T., Gunasekera S., Kummel D., Goransson U., Becker C.W., Gruber C.W. (2017). Chemical proteomics for target discovery of head-to-tail cyclized mini-proteins. Front. Chem..

[128] Austin J., Wang W., Puttamadappa S., Shekhtman A., Camarero J.A. (2009). Biosynthesis and biological screening of a genetically encoded library based on the cyclotide mcoti-i. ChemBioChem.

[129] Getz J.A., Cheneval O., Craik D.J., Daugherty P.S. (2013). Design of a cyclotide antagonist of neuropilin-1 and -2 that potently inhibits endothelial cell migration. ACS Chem. Biol..

[130] Maass F., Wustehube-Lausch J., Dickgiesser S., Valldorf B., Reinwarth M., Schmoldt H.U., Daneschdar M., Avrutina O., Sahin U., Kolmar H. (2015). Cystine-knot peptides targeting cancer-relevant human cytotoxic t lymphocyte-associated antigen 4 (ctla-4). J. Pept. Sci..

[131] Sharma P., Allison J.P. (2015). The future of immune checkpoint therapy. Science.

[132] Wang C.K., Stalmans S., De Spiegeleer B., Craik D.J. (2016). Biodistribution of the cyclotide mcoti-ii, a cyclic disulfide-rich peptide drug scaffold. J. Pept. Sci..

[133] Melander E., Eriksson C., Jansson B., Goransson U., Hammarlund-Udenaes M. (2016). Improved method for quantitative analysis of the cyclotide kalata b1 in plasma and brain homogenate. Biopolymers.

[134] Gao Y., Cui T., Lam Y. (2010). Synthesis and disulfide bond connectivity-activity studies of a kalata b1-inspired cyclopeptide against dengue ns2b-ns3 protease. Bioorg. Med. Chem..

[135] Wang C.K., Gruber C.W., Cemazar M., Siatskas C., Tagore P., Payne N., Sun G., Wang S., Bernard C.C., Craik D.J. (2014). Molecular grafting onto a stable framework yields novel cyclic peptides for the treatment of multiple sclerosis. ACS Chem. Biol..

[136] Chan L.Y., Gunasekera S., Henriques S.T., Worth N.F., Le S.J., Clark R.J., Campbell J.H., Craik D.J., Daly N.L. (2011). Engineering pro-angiogenic peptides using stable, disulfide-rich cyclic scaffolds. Blood.

[137] Swedberg J.E., Mahatmanto T., Abdul Ghani H., de Veer S.J., Schroeder C.I., Harris J.M., Craik D.J. (2016). Substrate-guided design of selective fxiia inhibitors based on the plant-derived momordica cochinchinensis trypsin inhibitor-ii (mcoti-ii) scaffold. J. Med. Chem..

[138] Chan L.Y., Craik D.J., Daly N.L. (2015). Cyclic thrombospondin-1 mimetics: Grafting of a thrombospondin sequence into circular disulfide-rich frameworks to inhibit endothelial cell migration. Biosci. Rep..

[139] Chan L.Y., Craik D.J., Daly N.L. (2016). Dual-targeting anti-angiogenic cyclic peptides as potential drug leads for cancer therapy. Sci. Rep..

[140] Slazak B., Kapusta M., Malik S., Bohdanowicz J., Kuta E., Malec P., Goransson U. (2016). Immunolocalization of cyclotides in plant cells, tissues and organ supports their role in host defense. Planta.

